# The Relationship Between Socio-Demographic Factors and Tuberculosis Mortality in the Republic of Korea During 2008–2017

**DOI:** 10.3389/fpubh.2021.691006

**Published:** 2021-10-20

**Authors:** SeoYeon Chung, Jeong-Yeon Seon, Seung Heon Lee, Hae-Young Kim, Yeo Wool Lee, Kyoungeun Bae, In-Hwan Oh

**Affiliations:** ^1^Department of Preventive Medicine, School of Medicine, Kyung Hee University, Seoul, South Korea; ^2^Division of Pulmonary, Sleep and Critical Care Medicine, Department of Internal Medicine Ansan, Korea University Ansan Hospital, Ansan-Si, South Korea; ^3^Department of Population Health, New York University Grossman School of Medicine, New York, NY, United States; ^4^Department of Public Health, School of Medicine, Korea University, Seoul, South Korea

**Keywords:** tuberculosis, TB-specific mortality, patient characteristics, TB incidence, TB control

## Abstract

The Republic of Korea has a high incidence of tuberculosis (TB) and TB-specific mortality rate. In 2019, it had the second highest TB-specific mortality among Organization for Economic Co-operation and Development countries. Understanding the factors associated with TB-specific deaths may help eradicate the disease. Therefore, we aimed to identify the general characteristics associated with TB-specific mortality among Koreans. Using Causes of Death Statistics data from Statistics Korea, we assessed the year of death, sex, age, occupation, area of residence, marital status, and education level reported between 2008 and 2017. Patient characteristics associated with TB-specific deaths were analyzed using the Chi-squared test, while influencing factors of TB-specific mortality were analyzed using logistic regression analysis to calculate adjusted odds ratios (AOR). Female (AOR: 0.509, 95% CI: 0.493–0.526), those with a graduate degree or higher (AOR: 0.559, 95% CI: 0.474–0.660) had lower TB-specific mortality rates than those of their counterparts. Conversely, those aged ≥70 years (AOR: 1.239, 95% CI: 1.199–1.280), single (AOR: 1.355, 95% CI: 1.315–1.396), and skilled agricultural, forestry, and fishery workers (AOR: 1.441, 95% CI: 1.359–1.529) had higher TB-specific mortality rates than those of their counterparts. In conclusion, TB-specific mortality rates differed according to the characteristics of the deceased patients. In order to establish effective TB control, multisectoral action on broader determinants should be strengthened.

## Introduction

Tuberculosis (TB) is an airborne infectious disease caused by *Mycobacterium tuberculosis* (MTB) (1). MTB has co-existed with humans for thousands of years (2). Approximately 1.7 billion people worldwide are suspected of being infected by MTB. In general, however, 90% of people infected with MTB maintain latent TB infection, and only 10% develop active TB over their lifetime (3, 4). MTB has characteristics that are different from other bacteria, and ~85% of TB cases can be treated with regular use of anti-TB drugs for at least 6 months (1, 5–7). Regarding TB treatment, medication compliance can be viewed as one of the most cost-effective interventions (8).

Since 1993, TB has been considered a global public health emergency (9). The TB-specific mortality rate has continued to decrease since 2000, but according to the 2020 Tuberculosis Report issued by the World Health Organization (WHO), 7.1 million people worldwide were newly diagnosed with TB in 2019, a slight increase from the 7.0 million people diagnosed in 2018. The report also indicated that 1.2 million deaths (range, 1.1–1.3) from TB were among HIV-negative people (1). Despite the fact that the global TB incidence has decreased by 9% between 2015 and 2019, it remains a major disease and one of the top 10 global causes of death by a single infectious agent (1).

There is evidence that TB is closely associated with socioeconomic indicators (10). The TB incidence is estimated to be ≤10 per 100,000 population per year in most high-income countries, whereas the rate is estimated to be 183 per 100,000 population per year in low- or middle-income countries (11). Studies have reported that people belonging to low socioeconomic groups have a higher risk of TB (12) and that there is a linear association between gross domestic product (13) per capita and the TB incidence rate (14). Moreover, other studies have reported that modifiable socio-economic risk factors could be potent factors of TB infection and disease (15). Among the socio-environmental and biological determinants of TB, the economic level has been recognized as a fundamental cause of TB (16, 17) due to its high prevalence among the poor (12), which makes the need to determine factors that influence the economic status even more evident. According to “Education at a Glance: OECD Indicators,” TB is highly prevalent among the poor and this may be attributable to the education level based on the annual reports on wage gaps by education levels (18).

The TB incidence rate in the Republic of Korea has decreased from 79 per 100,000 people in 2015 to 59 per 100,000 population in 2019, while the TB-specific mortality rate showed the largest decrease in 2019 (4.0 per 100,000 population) relative to 2015 (5.3 per 100,000 population) (5). Despite the continued decrease in incidence of TB and TB-specific mortality rates, the Republic of Korea has the highest TB incidence rate and second highest TB-specific mortality rate among the Organization for Economic Co-operation and Development (OECD) countries (1, 19). The incidence of TB and TB-specific mortality rates in the Republic of Korea remain very high compared with those of high-income countries, and the burden of disease must be reduced through national disease control efforts (20). Therefore, analyzing the factors that may influence TB-specific mortality could aid in preventing and controlling TB to ultimately eradicate it.

The characteristics that influence the incidence of TB have been analyzed in various countries and under various conditions (21–28). However, very few studies have compared the characteristics of deaths due to TB and other causes (29, 30) and, in particular, no studies have investigated such characteristics among Koreans. Accordingly, the objective of the present study was to use Population Trend Survey data from Statistics Korea to (1) compare the differences between deaths due to TB and other causes according to the characteristics of deceased patients and (2) analyze the characteristics of deceased patients that may influence TB-specific mortality to determine the factors that contribute to TB-specific mortality.

## Materials and Methods

In this cross-sectional study, we analyzed the characteristics of deceased patients that may influence TB-specific mortality based on deaths reported between 2008 and 2017. Data for deaths due to TB and other causes were obtained from the Causes of Death Statistics from Statistics Korea between 2008 and 2017. TB-specific mortality was defined according to the Korean Standard Classification of Disease and Cause of Death, 7th revision (KCD-7). The KCD-7 is a set of disease classification codes that were modified from the International Classification of Disease 10th revision (ICD-10) by WHO to reflect the situation in the Republic of Korea. In the present study, TB-specific mortality was defined as cause of death corresponding to KCD-7 code A15 (respiratory tuberculosis, bacteriologically and histologically confirmed), A16 (respiratory tuberculosis, not confirmed bacteriologically or histologically), A17 (tuberculosis of nervous system), A18 (tuberculosis of other organs), or A19 (military tuberculosis).

**Figure 1:**
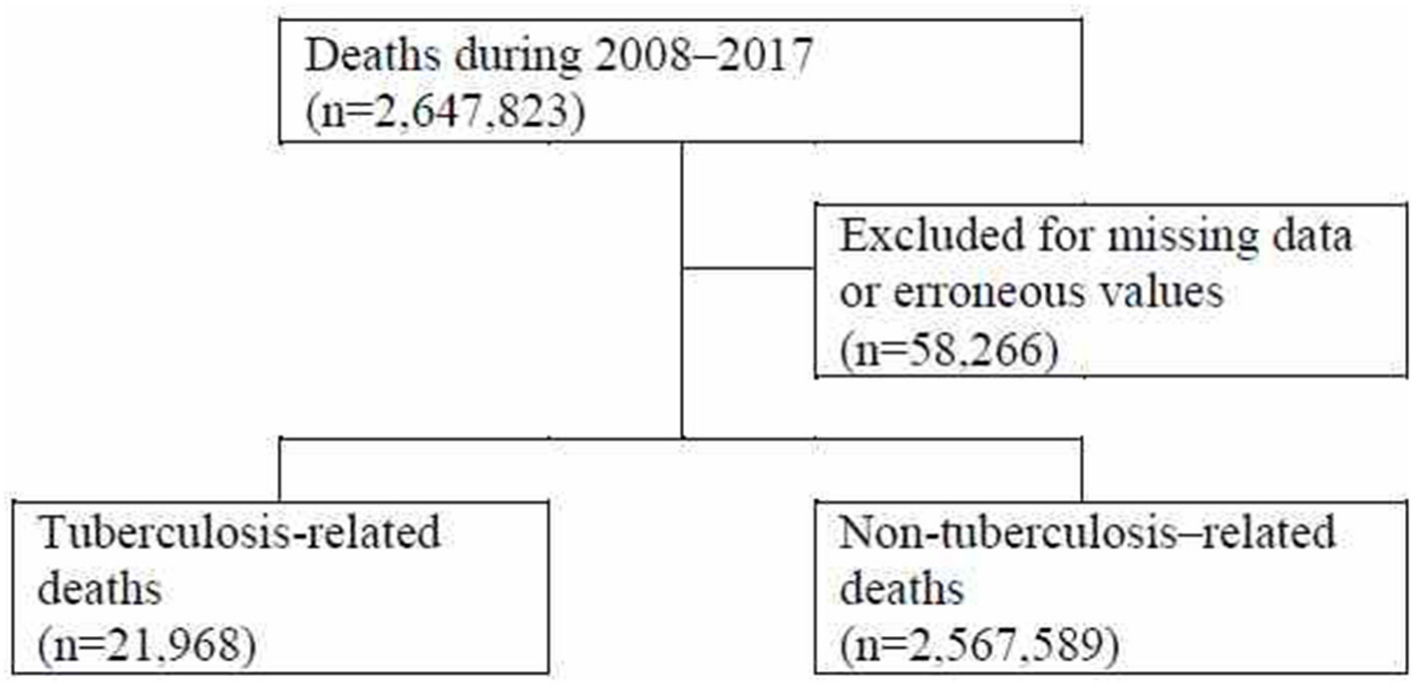
Flowchart of the analysis set selection process.

Among the 2,647,823 deaths reported between 2008 and 2017, cases with missing data and errors were excluded, resulting in a total of 2,589,557 deaths included in the analysis set. Of the 2,589,557 deaths in the analysis set, there were 21,968 deaths by TB and 2,567,589 deaths by other causes (Figure 1).

**Figure 2:**
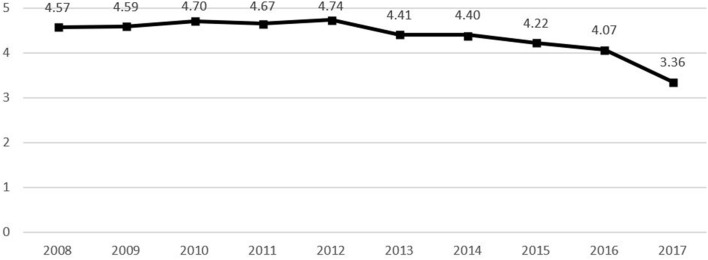
TB deaths per 100,000 population by years.

The time of death, sex, age, occupation, area of residence, marital status, education level, and place of death were considered in the analysis. TB deaths in Korea were declining between 2008 and 2017 (Figure 2), and year and month of death were examined as the time of death to identify TB-related deaths according to yearly trends and monthly changes. Typical demographic characteristics such as sex and age were considered as characteristics associated with deaths by TB, while age was divided into <65, 65–74, 75–84, ≥85 years to analyze the association between age and TB-related deaths. Occupation at the time of death was categorized into skilled agricultural, forestry, and fishery workers; students, homemakers, and unemployed; and others. Area of residence was divided into metropolitan and non-metropolitan areas. In accordance with the Enforcement Decree of the Seoul Metropolitan Area Readjustment Planning Act, Metropolitan areas included Seoul, Gyeonggi-do, and Incheon, while all other regions were defined as non-metropolitan areas. Moreover, marital status was divided into married and single, while education level was divided into primary school graduate or below; middle school graduate; high school graduate; college graduate; and graduate school or higher. Meanwhile, place of death was categorized into in-hospital and out-of-hospital to analyze its influence on TB-related death.

Chi-squared test and logistic regression analyses were used for statistical analysis. The Chi-squared test was performed to identify the characteristics of deceased patients that were associated with TB-related deaths, while a logistic regression model was established to analyze the characteristics of the deceased patients that influenced TB-related deaths. The logistic regression model was established by setting death due to TB as the outcome variable and year of death, sex, age, occupation, area of residence, marital status, and education level as the explanatory variables. SAS 9.4 (SAS Institute, Cary, NC, USA) was used for all statistical analyses and the significance level for all statistical testing was set to 5%.

The study was conducted with approval from the Korea University Medicine Institutional Review Board (IRB No. 2019AS0245).

## Results

The Chi-squared test was performed to analyze the associations between death from TB and deceased patient characteristics (year of death, season of death, sex, age, occupation, area of residence, marital status, education level, and place of death) (Table 1). All characteristics, except season of death, showed statistically significant results, while changes in trend for TB-specific mortality rate over time were identified for some variables.

**Table 1:** Socio-demographic factors of those with TB-specific deaths and non-TB–specific deaths. (Unit: deaths).

**Variables**		**Total**	**Cause of deaths**				***p*-value^**c**^**
			**TB-specific deaths**		**Non-TB–specific deaths**		
			***n* ^**a**^**	***%* ^**b**^**	**n^**a**^**	***%* ^**b**^**	
Total		2,589,557	21,968	100	2,567,589	100	<0.001
Year of death	2008 year	241,674	2,243	10.2	239,431	9.3	<0.001
	2009 year	243,922	2,265	10.3	241,657	9.4	
	2010 year	251,343	2,330	10.6	249,013	9.7	
	2011 year	254,163	2,331	10.6	251,832	9.8	
	2012 year	262,807	2,378	10.8	260,429	10.1	
	2013 year	262,974	2,222	10.1	260,752	10.2	
	2014 year	261,911	2,235	10.2	259,676	10.1	
	2015 year	269,887	2,154	9.8	267,733	10.4	
	2016 year	269,328	2,086	9.5	267,242	10.4	
	2017 year	271,548	1,724	7.8	269,824	10.5	
Month of death	January	224,762	1,888	8.6	222,874	8.7	0.153
	February	222,685	1,884	8.6	220,801	8.6	
	March	235,015	1,897	8.6	233,118	9.1	
	Aril	217,047	1,842	8.4	215,205	8.4	
	May	214,469	1,841	8.4	212,628	8.3	
	June	206,083	1,791	8.2	204,292	8	
	July	201,972	1,743	7.9	200,229	7.8	
	August	206,490	1,822	8.3	204,668	8	
	September	199,739	1,761	8	197,978	7.7	
	October	217,355	1,836	8.4	215,519	8.4	
	November	215,874	1,758	8	214,116	8.3	
	December	228,066	1,905	8.7	226,161	8.8	
Sex	Male	1,425,663	14,049	64	1,411,614	55	<0.001
	Female	1,163,894	7,919	36	1,155,975	45	
Age of death	<65 years	715,812	5,101	23.2	710,711	27.7	<0.001
	65–74 years	516,408	4,074	18.5	512,334	20	
	75–84 years	787,368	7,898	36	779,470	30.4	
	≥85 years	569,969	4,895	22.3	565,074	22	
Job	Skilled agricultural, forestry, and fishery workers	267,213	2,738	12.5	264,475	10.3	<0.001
	Students, homemakers, and unemployed	1,910,903	16,802	76.5	1,894,101	73.8	
	Other	411,441	2,428	11.1	409,013	15.9	
Residential area	Metropolitan	1,013,622	8,291	37.7	1,005,331	39.2	<0.001
	Non-metropolitan	1,575,935	13,677	62.3	1,562,258	60.8	
Marital status	Married	1,234,163	9,853	44.9	1,224,310	47.7	<0.001
	Single	1,355,394	12,115	55.1	1,343,279	52.3	
Education level	Primary school graduate or below	1,493,376	13,619	62	1,479,757	57.6	<0.001
	Middle school graduate	343,660	3,102	14.1	340,558	13.3	
	High school graduate	499,652	3,781	17.2	495,871	19.3	
	College graduate	222,251	1,320	6	220,931	8.6	
	Graduate school or higher	30,618	146	0.7	30,472	1.2	
Place of death	In-hospital	1,833,227	18,462	84	1,814,765	70.7	<0.001
	Out-of-hospital	756,330	3,506	16	752,824	29.3	

a *n, number of deaths*.

b *%, column percentages*.

c *p-value for Chi-squared test*.

The percentage of deaths from TB, according to the year of death, showed a decreasing trend. However, deaths from TB, according to the month of death, showed a similar distribution pattern, indicating no association. Regarding TB-specific deaths according to sex and age, the most frequent deaths were among male patients (64.0%, *n* = 14,049) and those aged 75–84 years (36.0%, *n* = 7,898). Statistical differences were found in gender and age distribution with non-tuberculosis deaths. Regarding TB-specific deaths according to occupation, the most frequent deaths were among students, homemakers, and unemployed subjects (76.5%, *n* = 16,802), followed by skilled agricultural, forestry, and fishery workers (12.5%. *n* = 2,738), and other occupations (11.1%, *n* = 2,428). In non-TB-specific deaths, the most frequent deaths were also among students, homemakers, and unemployed subjects (73.8%, *n* = 1,891,001). Moreover, the percentage of deaths from TB was high among non-metropolitan area residents (62.3%, *n* = 13,677) and among singles (55.1%, *n* = 12,115), while a decreasing trend in the percentage of deaths from TB was observed among patients with a higher education level. Finally, regarding the place of death, the percentage of in-hospital deaths from TB (84.0%, *n* = 18,462) was higher than that of out-of-hospital deaths by TB (16.0%, *n* = 3,506).

Logistic regression analysis was performed to analyze the characteristics of the deceased patients that influenced death from TB (Table 2). The logistic regression model included year of death, sex, age, occupation, area of residence, marital status, and education level as explanatory variables and statistical significance was confirmed in most explanatory variables. Cramer's V was reviewed to confirm the correlation between explanatory variables. All Cramer's vs. were <0.5, so we concluded that the logistic model had no multicollinearity. Regarding the Chi-squared test results, the percentage of deaths from TB tended to decrease when the time of death was more recent, and deaths from TB since 2013 showed statistically significant differences compared with 2008. TB as a cause of death was 0.509 times lower in female patients than in male patients (95% CI: 0.493–0.526) and 1.239 times higher in those aged ≥70 years than in those aged <70 years (95% CI: 1.199–1.280). Deaths among students/homemakers/unemployed and skilled agricultural/forestry/fishery workers were 1.427 times (95% CI: 1.363–1.495) and 1.441 times (95% CI: 1.359–1.529) higher, respectively, than in those with other occupations. Moreover, the crude odds ratio of death from TB according to area of residence was 1.121 (95% CI: 1.091–1.151), with a higher percentage among people who resided in non-metropolitan areas. The adjusted odds ratio was 1.000 (95% CI: 0.973–1.029), indicating no statistical significance. Death from TB was 1.355 times (95% CI: 1.315–1.396) higher in people who were single, while the percentage of deaths from TB tended to decrease as the education level increased. In particular, deaths in those with a graduate level education or higher was 0.559 times (95% CI: 0.474–0.660) lower than those who were primary school graduates or lower, showing an almost 56% decrease.

**Table 2:** Socio-demographic factors contributing to TB-specific deaths using logistic regression.

**Variables**		**Univariate model**		**Multivariate model**	
		**Crude OR^**a**^**	**95% CI^**b**^**	**Adjusted OR^**a**^**	**95% CI^**b**^**
Year of death	2008	1	1	1	1
	2009	1.001	(0.944–1.061)	1.002	(0.945–1.062)
	2010	0.999	(0.942–1.059)	0.997	(0.941–1.057)
	2011	0.988	(0.932–1.047)	0.985	(0.929–1.044)
	2012	0.975	(0.920–1.033)	0.969	(0.914–1.027)
	2013	0.91	(0.858–0.965)	0.906	(0.854–0.962)
	2014	0.919	(0.866–0.974)	0.917	(0.864–0.973)
	2015	0.859	(0.809–0.911)	0.856	(0.807–0.909)
	2016	0.833	(0.785–0.885)	0.833	(0.784–0.884)
	2017	0.683	(0.641–0.727)	0.682	(0.640–0.726)
Sex	Male	1	1	1	1
	Female	0.688	(0.670–0.708)	0.509	(0.493–0.526)
Age of death	<70	1	1	1	1
	≥70	1.282	(1.245–1.319)	1.239	(1.199–1.280)
Occupation	Other	1	1	1	1
	Students, homemakers, and unemployed	1.494	(1.432–1.559)	1.427	(1.363–1.495)
	Skilled agricultural, forestry, and fishery workers	1.744	(1.651–1.842)	1.441	(1.359–1.529)
Residential area	Metropolitan	1	1	1	1
	Non-metropolitan	1.062	(1.033–1.091)	1	(0.973–1.029)
Marital status	Married	1	1	1	1
	Single	1.121	(1.091–1.151)	1.355	(1.315–1.396)
Education level	Primary school graduate or below	1	1	1	1
	Middle school graduate	0.99	(0.952–1.029)	0.952	(0.913–0.992)
	High school graduate	0.828	(0.799–0.859)	0.836	(0.803–0.870)
	College graduate	0.649	(0.613–0.687)	0.656	(0.618–0.696)
	Graduate school or higher	0.521	(0.442–0.613)	0.559	(0.474–0.660)

a *OR, odds ratio*.

b *95% CI, 95% confidence interval*.

## Discussion

TB is a major cause of death and incurs a burden in low- and middle-income countries (31). Despite its high economic level, the Republic of Korea has a significantly high incidence of TB and mortality rates among OECD countries (1, 19), and still faces new challenges in terms of TB control. The present study aimed to compare the differences between deaths by TB and other causes according to the characteristics of the deceased patients and identify factors associated with TB-specific mortality rate.

In the present study, characteristics associated with TB-specific deaths were identified. The results showed that the TB-specific mortality rate is likely to be higher among male patients than female patients. Sex may be a factor that affects exposure to MTB due to differences in social roles and outdoor activities (32). Such results are consistent with most TB prevalence studies in countries with a high TB burden (33). Although the association between male sex and risk of TB is not clear, active TB is relatively more prominent among male patients (34), and there is almost no infectious disease that affects both sexes similarly (35). Despite this, TB is one of the top five causes of death among female patients and has a major impact on health in women (36). Moreover, analysis also showed that occupational factors influence TB-specific deaths. It is believed that agricultural, forestry, and fishery workers, which were some of the occupational sectors assessed, influenced TB-specific deaths since the percentage of men is high in such occupations. These findings agree with other studies reporting that men who have outdoor-labor occupations, such as mining, have a high risk of TB (37, 38). It is a long-held perception that TB is more common among the elderly population, and the findings of the present study are consistent with this (39). However, according to the 2020 Global Burden of Disease report, non-HIV related TB figures among the top 10 diseases affecting the global population aged 25–49 years (40). There were differences with previous patterns of TB-specific mortality or incidence rates, suggesting that TB is a disease that requires continued management regardless of age.

The findings of the present study showed no association between TB-specific death and season. These results are different from existing studies that reported that the incidence of TB around the world tends to be higher during spring or summer and lower during autumn or winter (41–47). In particular, TB notification rates in the Republic of Korea were lower during the winter, while summer or spring were the peak seasons for TB infection (48). Thus, the season may have an influence on the TB incidence rate but does not contribute to the mortality rate.

The socioeconomic status is determined by occupation, income, accumulated wealth, and education level (49). There are studies that measured the education level as a variable for assessing the socioeconomic status (50). The present study showed an association between a higher education level and lower percentage of TB-specific death. These results were similar to a study on all-cause mortality and risk factors in Taiwanese TB patients, which reported that the group who received high school education or higher had a lower risk of all-cause mortality than those who did not (51). While there are studies that reported a higher risk of TB in people with a higher education level (52), most studies have reported that patients with a lower education level lack an understanding of TB compared with patients with a higher education level (53), which often leads to discontinuation of treatment (54) and increased mortality (55). In particular, unlike other diseases, TB is a preventable (56, 57) and treatable disease (1, 58). It is necessary to implement systematic and continuous life education strategies to promote awareness and change the attitude toward TB prevention and control.

The WHO TB control strategy emphasizes that strategies for early detection and treatment completion by TB patients should be implemented (59). To achieve this, national level management is essential and the Korean government has been pursuing various TB control inventions over several decades (60). In 2013, the first National Strategic Plan for Tuberculosis Control (2013–2017) was established to enter a full-fledged TB eradication project (61). Subsequently, the second National Strategic Plan for Tuberculosis Control (2018–2022) was established, along with Measures to Strengthen TB Prevention/Management (2018–2022) with the goal of early TB eradication to lower the incidence of TB to 10 per 100,000 population by 2030 (62). The major strategies in the National Strategic Plan for Tuberculosis Control are target prevention and early detection of TB, patient treatment and contact management, expansion of TB-related research and development and management of essential goods, and strengthening of the TB eradication response system. Sub-tasks also include reinforcement and expansion of screening for the elderly, vulnerable populations, and high-risk groups with existing conditions. The policy is to eliminate blind spots in TB management by strengthening support and management for the elderly, homeless, and foreigners, and effectively achieve prevention, early diagnosis, and treatment by strengthening health insurance coverage for TB (63). Therefore, it is meaningful in that the incidence of TB by life cycle and target group can be reduced in all directions by strengthening the response system of government ministries and local governments.

The present study found that the TB-specific mortality rate decreased each year and the total number of new TB cases also showed a decreasing trend (62). According to the 2019 TB notification status report, the number of elderly patients (≥65 years) with TB decreased by 10.7% but the percentage of elderly patients among all new TB cases was 30.0, 45.5, and 47.1% in 2011, 2018, and 2019, respectively, and TB incidence rate was approximately 3.6 times higher among medical aid recipients than health insurance subscribers (5). Thus, the burden of TB among the elderly and socioeconomically vulnerable population remains. Despite being ranked as a high-income country with the tenth highest GDP in the world by the International Monetary Fund in 2019, the Republic of Korea has significantly high TB incidence and mortality rates among OECD countries. Therefore, the effects of the current policies appear to be insufficient, and the government should strive for quarantine measures and establishment of a sustainable and effective system for TB control.

According to the 2016 Tuberculosis Policy and Procedure Manual issued by the state of Georgia in the United States, homeless TB patient support and management is being implemented by confirmation of continued treatment and monthly review of patient care, along with budgetary support to provide shelter, including food and transportation costs, during the period for treatment completion and appropriate treatment (confirmation of medication compliance) (13). In Europe, a web-based TB NET is being operated for monitoring clinical and treatment outcomes of patients using an integrated clinical research collaboration among regions. In the United States, the Tuberculosis Genotyping Information Management System (TB GIMS) is being established by creating a database of whole genome sequencing of most reported cases of TB to forecast incidences of TB due to reactivation of MTB (64). Despite the low incidence of TB in the United States and Europe, a systematic and stable tuberculosis response system is in place. Considering the situation in Korea, it is necessary to establish an organized financial strategy and infrastructure to promote strong policies in Korea. In particular, since genome analysis using a database is useful during epidemiological investigations for analyzing the cause of infection and the spread of tuberculosis, it will be in demand to promote customized management policies through epidemiological characterization and environmental analysis.

The present study had some limitations. First, the data used in this study were complete enumeration survey data that were collected for establishing national policies and not for research purposes. However, the data were highly representative of all deaths. Therefore, the data were meaningful in that the characteristics of the target population could be estimated with minimal selection bias. Second, because only patients who died from TB were analyzed, the findings cannot be generalized for all TB patients. However, the present study was significant in that it differentiated deaths by TB from deaths by other causes. Lastly, the data used in the analysis were not adjusted for factors that have a definitive influence on TB-specific mortality, such as personal nutritional status, smoking status, diabetes, and HIV status (1). Despite this, the significance of the present study remains in the fact that it examined the characteristics of the deceased population that were associated with TB-specific mortality among Koreans.

Despite such limitations, the present study provides extensive epidemiological analysis data over 10 years on the TB-specific mortality rate in a country with a high burden of TB and identifies factors associated with TB-specific deaths and differences between characteristics. Moreover, the study presents aspects of TB control policies in the Republic of Korea that should be updated based on the findings of the study.

The present study used Causes of Death Statistics data from Statistics Korea to describe the percentage of TB-specific deaths reported between 2008 and 2017 in the Republic of Korea according to patient characteristics. This study also analyzed the factors associated with the TB-specific mortality to present aspects of TB control policies in the Republic of Korea that should be upgraded to reduce the mortality rate. Regarding the characteristics associated with the TB-specific mortality, year of death, sex, age, occupation, region of residence, marital status, education level, and place of death were identified as factors that may influence the mortality rate, but season did not show a significant association with TB-specific mortality. Accordingly, based on the findings of the present study, it is necessary to actively consider the positive aspects of TB control policies in other countries to use as a guide for implementing appropriate policies to eradicate TB in the Republic of Korea.

## Data Availability Statement

The original contributions presented in the study are included in the article/supplementary material, further inquiries can be directed to the corresponding authors. The raw data can be found at the Statistics Korea.

## Ethics Statement

The present study was conducted with approval from the Korea University Medicine Institutional Review Board (IRB No. 2019AS0245). Written informed consent for participation was not required for this study in accordance with the national legislation and the institutional requirements.

## Author Contributions

SC and J-YS: writing—original draft, conceptualization, data curation, formal analysis, validation, visualization, investigation, methodology, and writing—review & editing. I-HO: supervision, writing—review & editing, writing—original draft, and resources. SL: supervision, writing—review & editing, and resources. H-YK: supervision. YL: resources. KB: methodology. All authors contributed to the article and approved the submitted version.

## Funding

This research was supported by a grant of the Korea Health Technology R&D Project through the Korea Health Industry Development Institute (KHIDI), funded by the Ministry of Health & Welfare, Republic of Korea (Grant No. HI20C1068).

## Conflict of Interest

The authors declare that the research was conducted in the absence of any commercial or financial relationships that could be construed as a potential conflict of interest.

## Publisher's Note

All claims expressed in this article are solely those of the authors and do not necessarily represent those of their affiliated organizations, or those of the publisher, the editors and the reviewers. Any product that may be evaluated in this article, or claim that may be made by its manufacturer, is not guaranteed or endorsed by the publisher.

## References

[1] World Health Organization. Global Tuberculosis Report 2020. (2020). Available online at: https://www.who.int/publications/i/item/9789240013131 (accessed November 5, 2020).

[2] Comas I Coscolla M Luo T Borrell S Holt KE Kato-Maeda M. Out-of-africa migration and neolithic coexpansion of mycobacterium tuberculosis with modern humans. Nat Genet. (2013) 45:1176–82. 10.1038/ng.2744 23995134 PMC3800747

[3] Houben RM Dodd PJ. The global burden of latent tuberculosis infection: a re-estimation using mathematical modelling. PLoS Med. (2016)13:e1002152. 10.1371/journal.pmed.1002152 27780211 PMC5079585

[4] Jinsun K JeeYeon S JaeEun L Insik K. Review on Global Burden of Tuberculosis in 2018—Global Tuberculosis Report 2019. (2019). Available online at: http://www.kdca.go.kr/board/board.es?mid=a30501000000&bid=0031&list_no=365566&act=view# (accessed November 5, 2020).

[5] Korea Centers for Disease Control and Prevention. Annual Report on Notified Tuberculosis in Korea 2019. Cheongju-si: Korea Centers for Disease Control and Prevention (2020).

[6] Muture BN Keraka MN Kimuu PK Kabiru EW Ombeka VO Oguya F. Factors associated with default from treatment among tuberculosis patients in Nairobi province, Kenya: a case control study. BMC Public Health. (2011) 11:696. 10.1186/1471-2458-11-696 21906291 PMC3224095

[7] Furin J Cox H Pai M. Tuberculosis. Lancet. (2019) 393:1642–56. 10.1016/S0140-6736(19)30308-3 30904262

[8] Jamison DT Breman JG Measham AR Alleyne G Claeson M Evans DB editors. Disease control priorities in developing countries. 2nd ed. Washington (DC). In: The International Bank for Reconstruction and Development/The World Bank. New York, NY: Oxford University Press (2006). 10.1596/978-0-8213-6179-5 21250309

[9] Nakajima H. Tuberculosis: a global emergency. World Health. (1993) 46:3. 8185806

[10] Oxlade O Schwartzman K Behr MA Benedetti A Pai M Heymann J. Global tuberculosis trends: a reflection of changes in tuberculosis control or in population health? Int J Tuberc Lung Dis. (2009) 13:1238–46. 19793428

[11] World Health Organization. Global Tuberculosis Report 2018. (2018). Available online at: https://apps.who.int/iris/handle/10665/274453 (accessed November 5, 2020).

[12] Oxlade O Murray M. Tuberculosis and poverty: why are the poor at greater risk in India? PLoS ONE. (2012) 7:e47533. 10.1371/journal.pone.0047533 23185241 PMC3501509

[13] Georgia Department of Public Health. Tuberculosis Policy and Procedure Manual 2016. (2016). Available online at: https://dph.georgia.gov/document/document/tuberculosis-policy-and-procedure-manual-2016/download (accessed November 6, 2020).

[14] Janssens J-P Rieder HL. An ecological analysis of incidence of tuberculosis and per capita gross domestic product. Eur Respir J. (2008) 32:1415–16. 10.1183/09031936.00078708 18978146

[15] Saunders MJ Wingfield T Tovar MA Baldwin MR Datta S Zevallos K. A score to predict and stratify risk of tuberculosis in adult contacts of tuberculosis index cases: a prospective derivation and external validation cohort study. Lancet Infect Dis. (2017) 17:1190–99. 10.1016/S1473-3099(17)30447-4 28827142 PMC7611139

[16] Hargreaves JR Boccia D Evans CA Adato M Petticrew M Porter JD. The social determinants of tuberculosis: from evidence to action. Am J Public Health. (2011) 101:654–62. 10.2105/AJPH.2010.199505 21330583 PMC3052350

[17] Lönnroth K Castro KG Chakaya JM Chauhan LS Floyd K Glaziou P. Tuberculosis control and elimination 2010–50: cure, care, and social development. Lancet. (2010) 375:1814–29. 10.1016/S0140-6736(10)60483-7 20488524

[18] Organisation for Economic Co-operation and Development. Education at a Glance 2020: OECD Indicators. Paris: OECD Publishing (2020).

[19] Jinsun K JeeYeon S HyeKyung I Eunhye S. Review in Global Burden of Tuberculosis in 2019—Global Tuberculosis Report 2020. (2020). Available online at: http://www.kdca.go.kr/board/board.es?mid=a30501000000&bid=0031&list_no=710988&act=view# [accessed November 06, 2020].

[20] Kim JH Yim J-J. Achievements in and challenges of tuberculosis control in South Korea. Emerg Infect Dis. (2015) 21:1913–20. 10.3201/eid2111.141894 26485188 PMC4622233

[21] Gunal S Yang Z Agarwal M Koroglu M Arici ZK Durmaz R. Demographic and microbial characteristics of extrapulmonary tuberculosis cases diagnosed in Malatya, Turkey, 2001–2007. BMC Public Health. (2011) 11:154. 10.1186/1471-2458-11-154 21385458 PMC3060117

[22] Forssbohm M Zwahlen M Loddenkemper R Rieder HL. Demographic characteristics of patients with extrapulmonary tuberculosis in Germany. Eur Respir J. (2008) 31:99–105. 10.1183/09031936.00020607 17804450

[23] Nájera-Ortiz JC Sánchez-Pérez HJ Ochoa-Díaz H Arana-Cedeño M Lezama MS Mateo MM. Demographic, health services and socio-economic factors associated with pulmonary tuberculosis mortality in Los Altos Region of Chiapas, Mexico. Int J Epidemiol. (2008) 37:786–95. 10.1093/ije/dyn089 18511492

[24] Aghanwa H Erhabor GE. Demographic/socioeconomic factors in mental disorders associated with tuberculosis in southwest Nigeria. J Psychosom Res. (1998) 45:353–60. 10.1016/S0022-3999(98)00006-3 9794281

[25] Sampaio VS Rodrigues MGA Silva LCFD Castro DB Balieiro PCDS Cabrinha AA. Social, demographic, health care and co-morbidity predictors of tuberculosis mortality in Amazonas, Brazil: a multiple cause of death approach. PLoS ONE. (2020) 15:e0218359. 10.1371/journal.pone.0218359 31995562 PMC6988942

[26] Kipp AM Pungrassami P Nilmanat K Sengupta S Poole C Strauss RP. Socio-demographic and AIDS-related factors associated with tuberculosis stigma in southern Thailand: a quantitative, cross-sectional study of stigma among patients with TB and healthy community members. BMC Public Health. (2011) 11:675. 10.1186/1471-2458-11-675 21878102 PMC3223813

[27] Chan-yeung M Yeh AG Tam CM Kam KM Leung CC Yew WW. Socio-demographic and geographic indicators and distribution of tuberculosis in Hong Kong: a spatial analysis. Int J Tuberc Lung Dis. (2005) 9:1320–26. 16466053

[28] Mondal MN Nazrul HM Chowdhury MR Howard J. Socio-demographic factors affecting knowledge level of tuberculosis patients in Rajshahi City, Bangladesh. Afr Health Sci. (2014) 14:855–65. 10.4314/ahs.v14i4.13 25834494 PMC4379806

[29] Sterling TR Zhao Z Khan A Chaisson RE Schluger N Mangura B. Mortality in a large tuberculosis treatment trial: modifiable and non-modifiable risk factors. Int J Tuberc Lung Dis. (2006) 10:542–49. 16704037

[30] Hannah HA Miramontes R Gandhi NR. Sociodemographic and clinical risk factors associated with tuberculosis mortality in the United States, 2009-2013. Public Health Rep. (2017) 132:366–75. 10.1177/0033354917698117 28394707 PMC5415250

[31] World Health Organization. Global Tuberculosis Report 2013. Geneva: World Health Organization (2013) 289p.

[32] Nhamoyebonde S Leslie A. Biological differences between the sexes and susceptibility to tuberculosis. J Infect Dis. (2014) 209:S100–6. 10.1093/infdis/jiu147 24966189

[33] Hertz D Schneider B. Sex differences in tuberculosis. Semin Immunopathol. (2019) 41:225–37. 10.1007/s00281-018-0725-6 30361803

[34] Herzmann C Sotgiu G Bellinger O Diel R Gerdes S Goetsch U. Risk for latent and active tuberculosis in Germany. Infection. (2017) 45:283–90. 10.1007/s15010-016-0963-2 27866367 PMC5488071

[35] Guerra-Silveira F Abad-Franch F. Sex bias in infectious disease epidemiology: patterns and processes. PLoS ONE. (2013) 8:e62390. 10.1371/journal.pone.0062390 23638062 PMC3634762

[36] World Health Organization. Tuberculosis in Women. (2016). Available online at: https://www.who.int/tb/challenges/hiv/tb_women_factsheet.pdf (accessed November 5, 2020).

[37] Narasimhan P Wood J MacIntyre CR Mathai D. Risk factors for tuberculosis. Pulm Med. (2013) 2013:828939. 10.1155/2013/828939 23476764 PMC3583136

[38] Oni T Gideon HP Bangani N Tsekela R Seldon R Wood K. Smoking, BCG and employment and the risk of tuberculosis infection in HIV-infected persons in South Africa. PLoS ONE. (2012) 7:e47072. 10.1371/journal.pone.0047072 23056584 PMC3467259

[39] Schaaf HS Collins A Bekker A Davies PD. Tuberculosis at extremes of age. Respirology. (2010) 15:747–63. 10.1111/j.1440-1843.2010.01784.x 20546192

[40] GBD 2019 Diseases and Injuries Collaborators. Global burden of 369 diseases and injuries in 204 countries and territories, 1990–2019: a systematic analysis for the Global Burden of Disease Study 2019. Lancet. (2020) 396:1204–22. 10.1016/S0140-6736(20)30925-9 33069326 PMC7567026

[41] MacLachlan JH Lavender CJ Cowie BC. Effect of latitude on seasonality of tuberculosis, Australia, 2002–2011. Emerg Infect Dis. (2012) 18:1879. 10.3201/eid1811.120456 23092594 PMC3559156

[42] Khaliq A Batool SA Chaudhry MN. Seasonality and trend analysis of tuberculosis in Lahore, Pakistan from 2006 to 2013. J Epidemiol Global Health. (2015) 5:397–403. 10.1016/j.jegh.2015.07.007 26318884 PMC7320503

[43] Akhtar S Mohammad HG. Seasonality in pulmonary tuberculosis among migrant workers entering Kuwait. BMC Infect Dis. (2008) 8:3. 10.1186/1471-2334-8-3 18179720 PMC2259356

[44] Wubuli A Li Y Xue F Yao X Upur H Wushouer Q. Seasonality of active tuberculosis notification from 2005 to 2014 in Xinjiang, China. PLoS ONE. (2017) 12:e0180226. 10.1371/journal.pone.0180226 28678873 PMC5497978

[45] Margalit I Block C Mor Z. Seasonality of tuberculosis in Israel, 2001–2011. Int J Tuberc Lung Dis. (2016) 20:1588–93. 10.5588/ijtld.16.0306 28000582

[46] Willis MD Winston CA Heilig CM Cain KP Walter ND Mac Kenzie WR. Seasonality of tuberculosis in the United States, 1993–2008. Clin Infect Dis. (2012) 54:1553–60. 10.1093/cid/cis235 22474225 PMC4867465

[47] Korthals Altes H Kremer K Erkens C van Soolingen D Wallinga J. Tuberculosis seasonality in the Netherlands differs between natives and non-natives: a role for vitamin D deficiency? Int J Tuberc Lung Dis. (2012) 16:639–44. 10.5588/ijtld.11.0680 22410705

[48] Kim EH Bae J-M. Seasonality of tuberculosis in the Republic of Korea, 2006-2016. Epidemiol Health. (2018) 40:e201851. 10.4178/epih.e2018051 30486553 PMC6288684

[49] Kunst AE Mackenbach JP. Measuring socio-economic inequalities in health. Soc Sci Med. (1994) 44:757–71. 10.1016/S0277-9536(96)00073-1 9080560

[50] Álvarez JL Kunst AE Leinsalu M Bopp M Strand BH Menvielle G. Educational inequalities in tuberculosis mortality in sixteen European populations. Int J Tuberc Lung Dis. (2011) 15:1461–68. 10.5588/ijtld.10.0252 22008757 PMC3496173

[51] Yen YF Feng JY Pan SW Chuang PH Su VY Su WJ. Determinants of mortality in elderly patients with tuberculosis: a population-based follow-up study. Epidemiol Infect. (2017) 145:1374–81. 10.1017/S0950268817000152 28190404 PMC9203318

[52] Apolinário D Ribeiro AI Krainski E Sousa P Abranches M Duarte R. Tuberculosis inequalities and socio-economic deprivation in Portugal. Int J Tuberc Lung Dis. (2017) 21:784–89. 10.5588/ijtld.16.0907 28633703

[53] Hoa NP Diwan VK Co NV Thorson AE. Knowledge about tuberculosis and its treatment among new pulmonary TB patients in the north and central regions of Vietnam. Int J Tuberc Lung Dis. (2004) 8:603–8. 15137538

[54] Elzinga G Raviglione MC Maher D. Scale up: meeting targets in global tuberculosis control. Lancet. (2004) 363:814–19. 10.1016/S0140-6736(04)15698-5 15016493

[55] Okanurak K Kitayaporn D Akarasewi P. Factors contributing to treatment success among tuberculosis patients: a prospective cohort study in Bangkok. Int J Tuberc Lung Dis. (2008) 12:1160–65. 18812046

[56] Jung RS Bennion JR Sorvillo F Bellomy A. Trends in tuberculosis mortality in the United States, 1990–2006: a population-based case-control study. Public Health Rep. (2010) 125:389–97. 10.1177/003335491012500307 20433033 PMC2848263

[57] Velez DR Wejse C Stryjewski ME Abbate E Hulme WF Myers JL. Variants in toll-like receptors 2 and 9 influence susceptibility to pulmonary tuberculosis in Caucasians, African-Americans, and West Africans. Hum Genet. (2010) 127:65–73. 10.1007/s00439-009-0741-7 19771452 PMC2902366

[58] Nacher M Adenis A Aznar C Blanchet D Vantilcke V Demar M. How many have died from undiagnosed human immunodeficiency virus–associated histoplasmosis, a treatable disease? Time to Act Am J Trop Med Hyg. (2014) 90:193–94. 10.4269/ajtmh.13-0226 24277783 PMC3919218

[59] Lönnroth K Raviglione M. Global epidemiology of tuberculosis: prospects for control. Semin Respir Crit Care Med. (2008) 29:481–91. 10.1055/s-0028-1085700 18810682

[60] Cho KS. Tuberculosis control in the Republic of Korea. Epidemiol Health. (2018) 40:e2018036. 10.4178/epih.e2018036 30081621 PMC6335497

[61] Korea Ministry of Health and Welfare. The 1st Tuberculosis Management Comprehensive Plan. (2013). Available online at: https://www.mohw.go.kr/react/gm/sgm0704vw.jsp?PAR_MENU_ID=13&MENU_ID=13040801&page=1&CONT_SEQ=357091&PAR_CONT_SEQ=355753 (accessed November 7, 2020).

[62] Korea Ministry of Health and Welfare. The 2nd Tuberculosis Management Comprehensive Plan. (2018). Available online at: http://www.mohw.go.kr/react/jb/sjb030301vw.jsp?PAR_MENU_ID=03&MENU_ID=0319&CONT_SEQ=293110&page=1 (accessed November 7, 2020).

[63] Korea Ministry of Health and Welfare. Measures to Strengthen Tuberculosis Prevention and Management to Become a Nation Free From Tuberculosis. (2018). Available online at: http://www.mohw.go.kr/react/al/sal0301vw.jsp?PAR_MENU_ID=04&MENU_ID=0403&CONT_SEQ=349571&page=1 (accessed November 9, 2021).

[64] Korea Ministry of Health and Welfare. Research on Health Strategies for the Management of Tuberculosis in Developed Country-Style. (2018). Available online at: https://www.training.go.kr/pub/overseas/OutTrainResultReportDtl.do?trnCrsUid=111130&yr=2016&schdlNo=1&encUserUid=[e3]k1exCFZoCardBmm0bi9jk6HhErXA==&encRprtUid=[e3]YmaE0xbB77cT_K0ENkht7RffE6Fw==&encRprtSeq=[e3]5rXOIWPLGSCpfaYrHhD8AeuX-UCQ==&firstLoad=N (accessed November 7, 2020).

